# Interpretable and Reliable Oral Cancer Classifier with Attention Mechanism and Expert Knowledge Embedding via Attention Map

**DOI:** 10.3390/cancers15051421

**Published:** 2023-02-23

**Authors:** Bofan Song, Chicheng Zhang, Sumsum Sunny, Dharma Raj KC, Shaobai Li, Keerthi Gurushanth, Pramila Mendonca, Nirza Mukhia, Sanjana Patrick, Shubha Gurudath, Subhashini Raghavan, Imchen Tsusennaro, Shirley T. Leivon, Trupti Kolur, Vivek Shetty, Vidya Bushan, Rohan Ramesh, Vijay Pillai, Petra Wilder-Smith, Amritha Suresh, Moni Abraham Kuriakose, Praveen Birur, Rongguang Liang

**Affiliations:** 1 Wyant College of Optical Sciences, The University of Arizona, Tucson, AZ 85721, USA; 2 Computer Science Department, The University of Arizona, Tucson, AZ 85721, USA; 3 Mazumdar Shaw Medical Centre, Bangalore 560099, India; 4 KLE Society Institute of Dental Sciences, Bangalore 560022, India; 5 Mazumdar Shaw Medical Foundation, Bangalore 560099, India; 6 Biocon Foundation, Bangalore 560100, India; 7 Christian Institute of Health Sciences and Research, Dimapur 797115, India; 8 Beckman Laser Institute & Medical Clinic, University of California, Irvine, CA 92617, USA; 9 Cochin Cancer Research Center, Kochi 683503, India

**Keywords:** visual explanation, attention mechanism, human-in-the-loop deep learning, attention map, expert knowledge embedding, attention branch network

## Abstract

**Simple Summary:**

Convolutional neural networks (CNNs) have shown promising performance in recognizing oral cancer. However, the lack of interpretability and reliability remain major challenges in the development of trustworthy computer-aided diagnosis systems. To address this issue, we proposed a neural network architecture that integrates visual explanation and attention mechanisms. It improves the recognition performance via the attention mechanism while simultaneously providing interpretability for decision-making. Furthermore, our system incorporates Human-in-the-loop (HITL) deep learning to enhance the reliability and accuracy of the system through the integration of human and machine intelligence. We embedded expert knowledge into the network by manually editing the attention map for the attention mechanism.

**Abstract:**

Convolutional neural networks have demonstrated excellent performance in oral cancer detection and classification. However, the end-to-end learning strategy makes CNNs hard to interpret, and it can be challenging to fully understand the decision-making procedure. Additionally, reliability is also a significant challenge for CNN based approaches. In this study, we proposed a neural network called the attention branch network (ABN), which combines the visual explanation and attention mechanisms to improve the recognition performance and interpret the decision-making simultaneously. We also embedded expert knowledge into the network by having human experts manually edit the attention maps for the attention mechanism. Our experiments have shown that ABN performs better than the original baseline network. By introducing the Squeeze-and-Excitation (SE) blocks to the network, the cross-validation accuracy increased further. Furthermore, we observed that some previously misclassified cases were correctly recognized after updating by manually editing the attention maps. The cross-validation accuracy increased from 0.846 to 0.875 with the ABN (Resnet18 as baseline), 0.877 with SE-ABN, and 0.903 after embedding expert knowledge. The proposed method provides an accurate, interpretable, and reliable oral cancer computer-aided diagnosis system through visual explanation, attention mechanisms, and expert knowledge embedding.

## 1. Introduction

Convolutional neural networks have achieved outstanding performance in many visual tasks [1,2,3]. However, the end-to-end learning strategy used in CNNs makes them hard to interpret. It is difficult to fully understand the CNNs’ decision-making procedure that is hidden inside the network. Interpreting deep learning models has been a challenge for a long time. Many researchers have realized the significance and developed several methods for deep learning visual explanation [4]. Visual explanation generates an attention map that highlights discriminative regions used for CNN decision-making, which is a common approach for interpreting deep learning models. There are two types of visual explanations: response-based and gradient-based. Response-based approaches, such as Class Activation Mapping (CAM) [5], use the response of the convolutional layer to generate the attention map. Gradient-based approaches, such as gradient weighted-CAM (Grad-CAM) [6], use gradient and feed forward response to generate the attention map. CAM and Grad-CAM are two widely used visual explanation methods. CAM uses the K channel feature map from the convolution layer and the weight at a fully connected layer to calculate the attention map. However, this method requires modification of the CNN architectures, that is, replacing the fully connected layer of the original network with a convolutional layer and global average pooling. Grad-CAM uses the response of the convolution layer and a positive gradient in the backpropagation process to generate the attention map. Grad-CAM can be applied to interpret various models without changing network architecture or re-training.

Attention mechanism is a powerful tool that efficiently allocates the available processing resources to the most informative part of the input signal [7]. It has been applied to many fields such as computer vision and natural language processing. The attention mechanism is usually implemented in combination with a gating function such as softmax or sigmoid and sequential techniques. In image recognition tasks, previous researchers have proposed several attention-based approaches. One such approach is Squeeze-and-Excitation network (SENet) [8], which allows the network to perform feature recalibration. It can use the global information to emphasize the most informative features and suppress the less informative ones. The SE block is a lightweight gating mechanism that models channel-wise relationships in a computationally efficient manner. Another approach is Residual Attention Network [9], which employs multiple attention modules, each with a mask branch and a trunk branch. It also utilizes an attention residual learning mechanism to optimize very deep Residual Attention architecture and bottom-up top-down feedforward attention structure.

Attention branch network (ABN) [10], inspired by visual explanation and attention mechanisms, uses the attention map for both visual explanation and attention mechanism. The highlighted region in the attention map is considered an informative part and obtains more attention in image recognition. ABN has a feature extractor to extract features; the feature extractor could be various baseline models such as Resnet or VGGNet. It also consists of an attention branch and a perception branch. The attention branch extends the response-based visualization method CAM to generate an attention map. The perception branch of the ABN model utilizes the informative regions and highlighted regions in the attention map to emphasize the relevant features and suppress others to produce the final results. By integrating visual explanation and attention mechanism, the ABN model can interpret the decision-making of the deep learning network and improve the recognition performance simultaneously. Ding et al. [11] proposed a deep attention branch network by introducing two attention branches into a baseline model composed of four dense blocks, three transition layers, and a classification layer. Additionally, an entropy-guided loss weighting strategy was introduced to address the class imbalance problem. The experimental results demonstrate that the proposed method can improve the focusing ability of networks to accurately locate the discriminative lesion regions and improve the classification performance; the entropy-guided loss weighting strategy can further boost the performance.

Human-in-the-loop (HITL) deep learning [12,13] is a set of strategies that integrates human knowledge and machine intelligence to enhance the performance of deep learning models. HITL has attracted significant research interest in the machine learning community, and many studies have investigated this topic by leveraging the complementary strengths of human and machine intelligence, resulting in improved accuracy compared to machine intelligence alone. For instance, Zhu et al. [14] proposed a tool that integrates human physicians’ knowledge and deep learning algorithms for efficient object detection of renal pathology. Linsley et al. [15] developed a ClickMe map that collects human feedback to train the deep learning model via the HITL framework. The method achieved better performance by introducing human knowledge to the weight of the attention mechanism. Mitsuhara et al. [16] used manually editable attention maps to embed human knowledge into deep neural networks. Human experts can intuitively understand the attention map and edit it interactively through a visual interface. The edited attention maps can improve recognition performance by reflecting human knowledge.

Oral cancer is one of the most common cancers worldwide and is the second most common cancer in India [17]. Most high-risk populations living in low- and middle-income countries do not have adequate medical resources for early diagnosis and treatment. Therefore, researchers have developed cost-effective methods for oral cancer diagnosis such as fluorescence imaging [18] and fluorescence lifetime imaging [19] to meet these pressing needs, and these methods have been successfully implemented in low-resource settings. For instance, Duran-Sierra et al. [19] developed and validated a machine-learning assisted computer aided detection system to automatically differentiate dysplastic and cancerous tissue from healthy oral tissue based on in vivo widefield autofluorescence lifetime imaging endoscopy data. This study evaluated four traditional machine learning models and did not use convolutional neural network models. Convolutional neural networks are powerful tools in medical image analysis, and multiple deep learning-based oral cancer recognition approaches have been introduced [20,21,22,23]. However, improving the accuracy, reliability, and interpretability of these models is still challenging. In this work, we use the attention branch network and Squeeze-and-Excitation blocks to apply visual explanation and attention mechanisms into the oral cancer recognition model. The attention map generated from the attention branch can interpret the model’s predictions and improve the performance through the perception branch via the attention mechanism. Additionally, human experts manually edited the automatically generated attention map and fed it back to the network’s perception branch. The manual editing helps to accurately highlight the oral lesion or healthy regions according to the annotation of oral oncology specialists. Our experimental results demonstrate that incorporating ABN and SE blocks improves the classification accuracy of convolutional networks. Furthermore, expert knowledge, in the form of manually edited attention maps, leads to improved reliability and performance.

## 2. Materials and Methods

In Section 2.1, we introduce the Attention branch network (ABN) and discuss its two main components: the attention branch and the perception branch (Section 2.1.1 and Section 2.1.2). We then outline the training process for ABN in Section 2.2. In Section 2.3, we discuss how human expert knowledge can be integrated into the ABN network to improve its performance.

Additionally, in Section 2.4, we present another attention method, the Squeeze-and-Excitation, which we used to further enhance the network’s performance. Finally, we describe the dataset used for this study in Section 2.5.

### 2.1. Attention Branch Network

**Figure 1:**
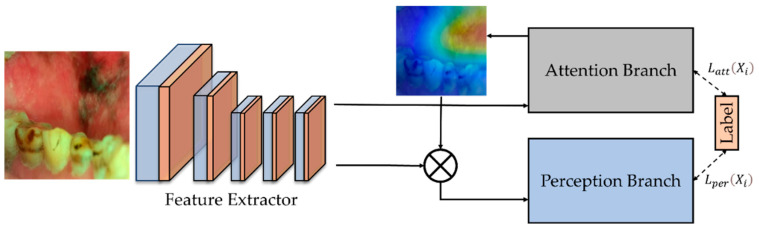
The block diagram of the attention branch network for our oral cancer task [10]. It has a feature extractor, an attention branch, and a perception branch. The perception branch uses the attention map generated from the attention branch to emphasize the most informative features.

Attention branch network (ABN) [10] extends the response-based visual explanation model, which is able to visualize the attention map for visual explanation while improving the CNN performance with the attention mechanism simultaneously. It consists of three components: the feature extractor that contains convolutional layers to extract feature maps from the input image; the attention branch that generates an attention map based on CAM for the attention mechanism and visual explanation; and the perception branch that outputs the probabilities of classes using the feature map from feature extractor and attention map from the attention branch. The block diagram of the attention branch network for our oral cancer classification task is shown in Figure 1.

#### 2.1.1. Attention Branch

The ABN extends the CAM. CAM applies global average pooling (GAP) on the convolutional feature maps to produce the desired output. It can identify the importance of the image areas for CNN decision-making by projecting back the weights of the output layer onto the convolutional feature maps. When CAM visualizes the attention map of each class, the attention map is generated by multiplying the weighted sum of the feature map. CAM removes the fully connected layers before the final output and replaces them with convolution layers. Then, it adds a GAP and a fully connected softmax layer. This fully connected layer replacement restriction is also introduced into the attention branch. Similar to CAM, the attention branch uses convolution layer and GAP to generate an attention map. However, the attention branch replaces the fully connected layer with a Kx1x1 convolution layer (K is the number of categories) since CAM cannot generate an attention map in the training process. The Kx1x1 convolution layer imitates the last fully connected layer of CAM. The class probability output is generated using the response of GAP with the softmax function after the Kx1x1 convolution layer. The attention branch also generates an attention map for the attention mechanism. The K feature maps are convoluted by a 1x1x1 convolution layer and then normalized by the sigmoid function as the attention map.

#### 2.1.2. Perception Branch

The perception branch outputs the classification results using the attention maps from the attention branch and feature maps from the feature extractor with an attention mechanism. In this study, the attention map $M\left(X_{i}\right)$ is applied to the feature map $g_{c}\left(X_{i}\right)$ by the following attention mechanism:

$$g\prime_{c}\left(X_{i}\right)=g_{c}\left(X_{i}\right)\cdot M\left(X_{i}\right)$$

### 2.2. Training of ABN

The loss function of ABN jointly optimizes both attention and perception branches. The combined loss function $L\left(X_{i}\right)$ was constructed as:

$$L\left(X_{i}\right)=L_{att}\left(X_{i}\right)+L_{per}\left(X_{i}\right)$$

where $L_{att}\left(X_{i}\right)$ is the attention branch training loss and $L_{per}\left(X_{i}\right)$ is the perception branch training loss. The training loss of each branch is calculated by the combination of the softmax function and cross-entropy.

### 2.3. Manual Editing of Attention Map

**Figure 2:**
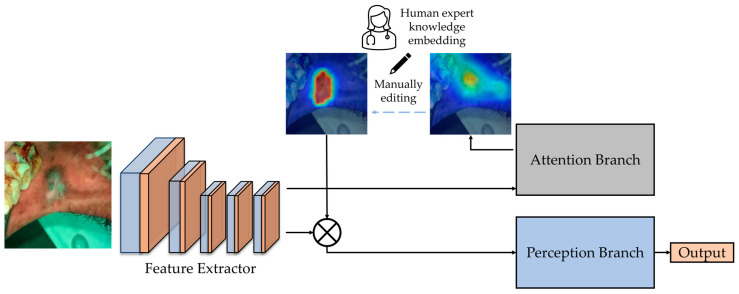
The block diagram shows the embedding of expert knowledge into the network [16]. The attention maps generated from the attention branch were manually edited and sent back to emphasize the most informative features.

As mentioned before, in ABN, the attention map generated from the attention branch is used for the attention mechanism. The classification result could be adjusted by editing the attention map. To manually edit the attention map, one initial attention map was obtained from the attention branch of a trained ABN. Then an attention editor [16] can be used to manually edit the obtained attention maps interactively. The attention editor is created using PyQt5 [24] and PyTorch, which can add and remove an attention region easily via mouse. Since the size of the attention map generated from the attention branch is 14 × 14 pixels, the attention editor resizes it to 224 × 224 pixels and overlays it with the input oral image. After editing, the edited attention map is resized to 14 × 14 pixels, and the tool feeds it back for the attention mechanism of ABN to infer updated classification results through the perception branch. By highlighting the attention location of lesion areas and removing other regions on the attention map, the edited attention map can improve the classification results through the attention mechanism of ABN. The block diagram of the expert knowledge embedding is shown in Figure 2.

### 2.4. SENet

In this study, Resnet18 was used as the baseline network to implement ABN. To further improve the performance of the Resnet18-ABN network for the oral cancer classification task, Squeeze-and-Excitation (SE) blocks were also incorporated into the network. SE block introduces a channel attention mechanism that is composed of three components: squeeze module, excitation module, and scale module.

The squeeze module uses global average pooling to generate channel-wise statistics, which reduces the feature map to a single value by taking the average of all the pixels in that feature map. If the input feature maps size is CxHxW, the output tensor will be Cx1x1 after passing through the GAP operation. Each feature map is decomposed into a singular value. The excitation module is to learn the adaptive scaling weights for the Cx1x1 tensor generated from the squeeze module. A gating mechanism with a sigmoid activation is employed. The gating mechanism is parameterized by forming a bottleneck with two fully connected layers, a dimensionality-reduction layer, a ReLU, and a dimensionality-increasing layer. The excitation module inputs the Cx1x1 tensor and outputs a weighted tensor of the same Cx1x1 size. After obtaining the Cx1x1 weighted tensor from the excitation module, it is scaled to a range of 0–1 through a sigmoid activation layer. Subsequently, the normalized weighted tensor is applied directly to the input by an element multiplication that scales each channel/feature map in the input with the corresponding learned weights.

The SE block could be applied to multiple existing network architectures and improves the network performance at a minimal additional computational cost. When adding the SE block to the residual network, it is inserted after the final convolutional layer of the residual block and before the residual is added to the skip connection.

### 2.5. Dataset

The dataset used in this study was captured using our customized oral cancer screening platform [25], which was obtained from patients attending the outpatient clinics of the Department of Oral Medicine and Radiology at the KLE Society Institute of Dental Sciences (KLE), the Head and Neck Oncology Department of Mazumdar Shaw Medical Center (MSMC), and the Christian Institute of Health Sciences and Research (CIHSR), India. Institutional ethics committee approval was obtained from all participating hospitals and written informed consents were collected from all subjects enrolled.

The data collection and study followed the International Conference of Harmonization recommendation on Good Clinical Practice, and all methods were carried out in accordance with relevant guidelines and regulations. The study protocol was registered in the Clinical Trial Registry of the Indian Council of Medical Research (CTRI/2019/11/022167, Registered on: 27 November 2019). The subjects were recruited at the study sub-centers, which were monitored by nodal centers in a hub-and-spoke model. Institutional Ethics Committee approvals were obtained from all nodal centers. The participants who were above 18 years of age, with a history of tobacco smoking and/or chewing, or with any oral lesion were included, and written informed consent was obtained from all the participants. The individuals currently undergoing treatment for malignancy, pregnancy, tuberculosis, or suffering from any acute illness were excluded. All the subjects included in the study were directly telediagnosed by remote specialists [26].

**Figure 3:**
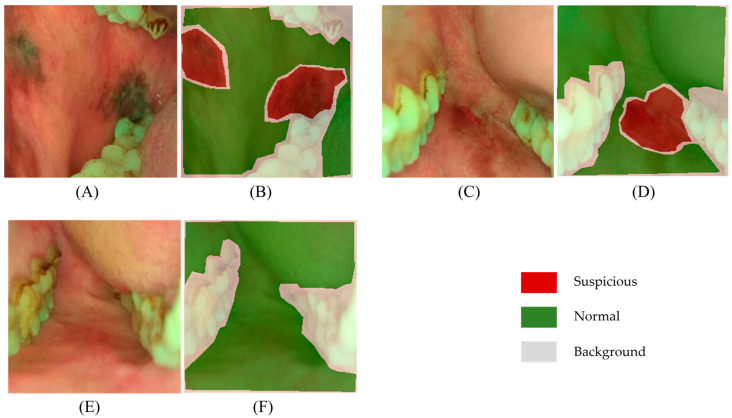
Examples of the dataset and oncology specialists’ annotations. (**A**,**C**,**E**) are white light oral cavity images captured using our customized oral cancer screening platform. (**B**,**D**,**F**) are corresponding pixel-level annotations labeled by oral oncology specialists. The oral potentially malignant lesion and malignant lesion areas are shown in red, normal and benign areas are shown in green, and other background areas are shown in grey.

We used a total of 2040 oral images to validate this method for oral cancer classification. The images were separated into two categories: ‘normal’ (978 images), which contains normal and benign mucosal lesion images, and ‘suspicious’ (1062 images), which contains oral potentially malignant lesion (OPML) and malignant lesion images. The oral lesion regions for attention map editing were based on oral oncology specialists’ annotations from MSMC, KLE, and CIHSR. In a previous study, we showed that oral oncology specialists’ interpretation of classifying normal/benign versus OPML/malignant has high accuracy with biopsy-confirmed cases [27]. Examples of the dataset used in this study and the oncology specialists’ annotations is shown in Figure 3.

## 3. Results

In this study, all experiments were conducted using five-fold cross-validation. The networks were trained using the cross-entropy loss and the Adam optimization algorithm that were implemented on PyTorch. Data augmentation was applied to the training set by flipping horizontally and vertically, random rotating, and shearing while training all networks. For each training, the initial learning rate was 10^−3^, which decayed 10 times by every 50 epochs, and the epoch number was 180 with a batch size of 32. We saved the models with the best validation accuracy.

In the first set of experiments, we trained the attention branch network and ABN with SE blocks (SE-ABN) using different baseline networks, including Resnet18, Resnet34, Resnet50, and Resnet101, to verify whether the method could improve the oral cancer classification performance. We also trained the original Resnet18, Resnet34, Resnetfive0, and Resnet101 networks with the same data and parameters for comparison purposes. Table 1 shows the five-fold cross-validation results of these experiments. Our findings show that ABN outperforms the original baseline network, and by introducing the SE blocks to ABN, the cross-validation accuracy is further increased. These results indicate that ABN can help the network pay more attention on lesion regions, leading to improved accuracy, and SE blocks can further improve the performance through its channel attention mechanism.

In addition, we observed that although deeper Resnet models have more layers and require more computational time and resources, the performance difference on this oral dataset is not significant. Therefore, for the next set of attention map experiments, we will use ABN and SE-ABN with the Resnet18 baseline network.

### 3.1. Visualizing Attention Maps

**Figure 4:**
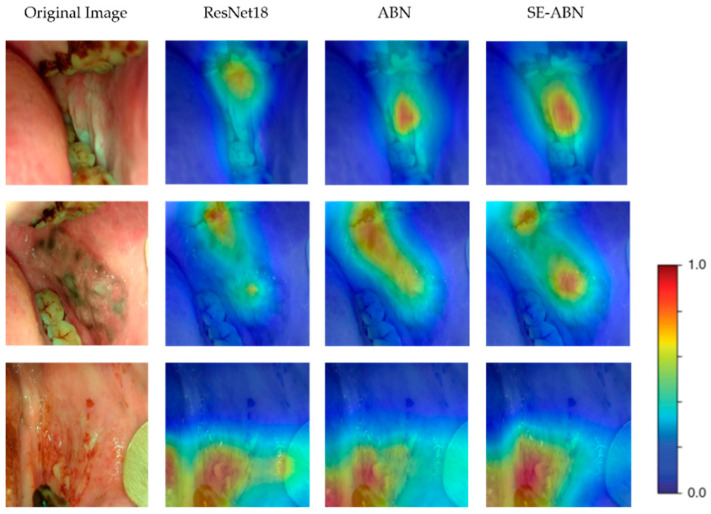
The attention maps of three example cases were repectively generated from the original Resnet18, ABN, and SE-ABN. The first column shows the original oral images, the second column shows the attention maps generated from the original Resnet18, the third column shows the attention maps generated from ABN (Resnet18 baseline), and the fourth column shows the attention maps generated from SE-ABN (Resnet18 baseline). Although all three models highlight similar regions, the attention maps of SE-ABN are more accurate than those of ABN in identifying the lesion areas, and both SE-ABN and ABN focused more accurately than the original Resnet18 network. For instance, in the first and second images, the attention maps of the original Resnet18 focused more on teeth than the lesion area, whereas SE-ABN clearly highlighted the lesion areas.

To compare the attention maps generated by different models, we used three example cases and visualized the attention maps of the original Resnet18, ABN, and SE-ABN. The results are shown in Figure 4. While all three models highlight similar regions, the attention maps of ABN and SE-ABN are more accurate than the original Resnet18 in identifying the lesion areas. For the first and second images, the attention maps of the original Resnet18 focused more on teeth than the lesion area, while ABN and SE-ABN focused more accurately on the lesion when making decisions. The results indicate that the attention mechanism of ABN and SE block can help the network effectively focus on the lesion regions instead of background areas such as teeth. The mismatch between the classification and the attention region could degrade the reliability of the model performance, especially for medical image recognition systems. Therefore, ABN and SE-ABN networks are more reliable since they can focus more accurately on lesion areas for decision making.

### 3.2. Incorporating Manually Edited Attention Maps

In this experiment, we employed the SE-ABN with Resnet18 backbone for attention map editing, with the aim of improving the classification performance.

To perform attention map editing, we followed the procedure outlined in Section 2.2, which involved inputting each validation image to the model and obtaining the attention map from the attention branch. The generated attention map would be overlaid with the input oral image for manual editing using the attention editor tool. Then the human experts used the editor tool to add and remove attention regions to ensure that the edited attention maps accurately and completely highlighted the corresponding regions. Finally, we sent the edited attention maps back to the attention mechanism of SE-ABN to obtain updated classification results through the perception branch.

**Figure 5:**
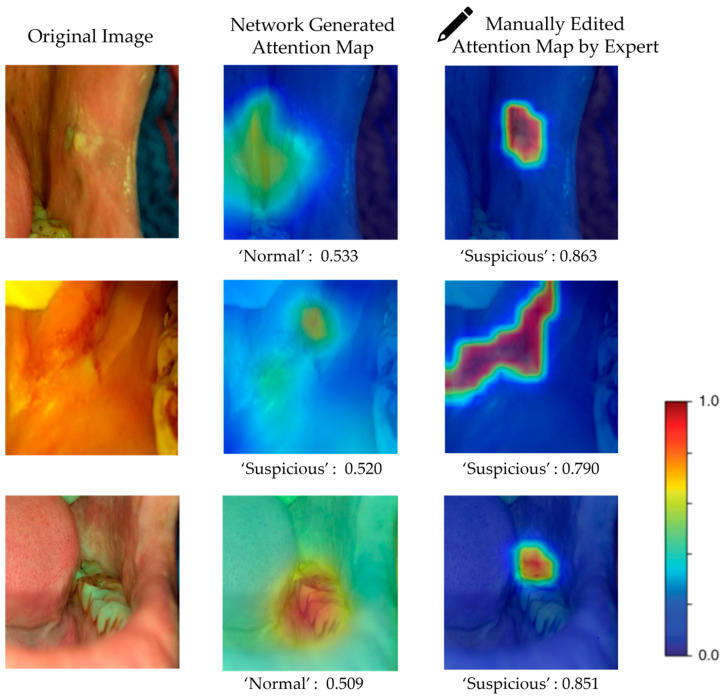
Three examples of manually edited attention maps, and the corresponding results before and after embedding human expert knowledge. The class label here means prediction, and the number after means the probability score. The first and third examples show that previous misclassified cases were correctly recognized after manually editing the attention maps by highlighting the lesion regions accurately and completely. The network failed to give correct predictions or focus correctly on lesion areas for the first and third cases, but after manually editing to let the network look at the accurate areas, the correct predictions were presented. Although the network gave a correct prediction for the second case, the probability score is low, while the probability score for the ‘suspicious’ class of the second case increased after editing.

Figure 5 presents several examples of attention map editing. In the first and third examples, the original attention maps obtained from the attention branch were incomplete and inaccurate in highlighting the lesion areas, resulting in a false classification of ‘normal’ for both cases. However, after manually editing the attention maps using the attention editor tool, the lesion areas were accurately and completely highlighted. The updated attention maps were then used for the attention mechanism of the model, resulting in a correct classification of ‘suspicious.’ Similarly, in the second example, although the model classifies the input image correctly as ‘suspicious’ with a probability score of 0.520, the attention map obtained from the attention branch did not completely highlight the lesion region. After manually editing the attention map, the probability score increased to 0.790. These results demonstrate that the attention map editing process can recognize more lesion features after highlighting more accurate and complete areas.

The five-fold cross-validation accuracy of SE-ABN before and after editing the attention maps is shown in Table 2. The attention map editing process resulted in an increase in the validation accuracy from 87.7% to 90.3%. These results indicate that by editing the attention maps to highlight the accurate and complete lesion or normal areas, the network can focus on these areas via the attention branch and recognize more accurate features, resulting in improved classification accuracy.

## 4. Discussion

The experimental results of our proposed model have demonstrated a higher classification accuracy compared to baseline models. Additionally, the visual explanation results have shown that our proposed model can identify the lesion areas more accurately when making decisions. These results provide evidence that our proposed method improves the interpretability and reliability of the model via attention mechanism and visual explanation and successfully embeds human knowledge for the oral cancer recognition task.

The use of visual explanation and more accurate attention maps in the proposed AI model improves the model’s reliability. By visualizing the areas that the model is focusing on during decision-making, we can observe whether the model is looking at correct/accurate lesion areas in addition to the classification results. The increased sensitivity and specificity makes the model more effective in cancer screening, as false positives can lead to unnecessary psychological stress, medical procedures, and increased clinical workloads. Furthermore, the manually edited attention maps generated by human experts have the potential to aid in the localization of biopsies. By highlighting the regions of interest with high accuracy and completeness, these attention maps can be used by on-site doctors to better locate biopsies.

Incorporating human expert knowledge into the decision-making process can enhance the accuracy and reliability of computer aided diagnosis system. In conjunction with our previously developed uncertainty assessment method [23], we could integrate the human expert knowledge into cases that Bayesian deep learning model is uncertain. This approach is not limited to oral cancer diagnosis, and we think any image-based cancer diagnosis approach that requires identification of the lesion areas can potentially benefit from this method.

## 5. Conclusions

Deep learning is a powerful tool in solving medical image analysis tasks. However, interpretability and reliability remain as challenges. In this study, we used an attention branch network for the oral cancer recognition task; it combines visual explanation and attention mechanism. The network can simultaneously interpret the decision-making and improve the recognition performance using the attention map with an attention mechanism. The attention branch of the network extends the response-based visualization method and generates an attention map, and then the perception branch uses the attention map to emphasize the most informative features extracted by the feature extractor of the network.

The attention mechanism has been widely used and has demonstrated exceptional performance in various deep learning tasks [7]. In previous attention models, the weights for the attention mechanism were obtained solely from the response value of the convolution layers during feed forward propagation in an unsupervised learning manner. However, ABN extracts the weight for an attention mechanism in image recognition by generating the attention map for visual explanation on the basis of response-based visual explanation in a supervised learning manner [10]. With ABN, the cross-validation accuracy of the oral image dataset improved to 0.875 from 0.846. After applying another attention method, the Squeeze-and-Excitation block, the accuracy further boosted to 0.877. It enables the network to perform dynamic channel-wise feature recalibration. Additionally, we incorporated the expert knowledge into the network by manually editing the attention map generated from the attention branch. The edited attention maps were then fed back into the network’s perception branch and which updated the result via the attention mechanism. As a result, the cross-validation accuracy of the oral image dataset achieved 0.903.

The experiment’s results have shown that the attention branch network and Squeeze-and-Excitation block can effectively improve the recognition performance as well as interpret the decision-making. Further, embedding the expert knowledge led to a further increase in accuracy. The proposed method provided an accurate, interpretable, and reliable oral cancer classifier that leverages visual explanation, attention mechanisms, and human expert knowledge embedding.

## Figures and Tables

**Table 1:** The five-fold cross-validation accuracy of ABN, SE-ABN, and the original network with different baseline networks.

Five-Fold Cross-Validation Accuracy	ResNet18	ResNet34	ResNet50	ResNet101
Original Network	0.846	0.851	0.850	0.844
ABN	0.875	0.879	0.880	0.872
SE-ABN	0.877	0.880	0.881	0.876

**Table 2:** The performance comparison of the original network, ABN, SE-ABN, and manually edited attention maps.

Original ResNet18 network	Sensitivity	0.833
	Specificity	0.857
	Positive predictive value	0.843
	Negative predictive value	0.848
	Accuracy	0.846
ABN	Sensitivity	0.860
	Specificity	0.887
	Positive predictive value	0.876
	Negative predictive value	0.873
	Accuracy	0.875
SE-ABN	Sensitivity	0.868
	Specificity	0.886
	Positive predictive value	0.875
	Negative predictive value	0.879
	Accuracy	0.877
SE-ABN (incorporating manually edited attention maps)	Sensitivity	0.898
	Specificity	0.908
	Positive predictive value	0.899
	Negative predictive value	0.906
	Accuracy	0.903

## Data Availability

Data underlying the results presented in this paper are not publicly available.
